# Utility of Serum Matrix Metalloproteinase-7 as a Biomarker in Cholestatic Infants with Congenital Heart Disease

**DOI:** 10.21203/rs.3.rs-5004969/v1

**Published:** 2024-10-18

**Authors:** Sindhu Pandurangi, Michael E. Kim, Nicolas Noriega, Bradley Conant, JangDong Seo, Reena Mourya, Pranavkumar Shivakumar, Anna L. Peters, Andrew Misfeldt, Meghan Chlebowski

**Affiliations:** University of Texas Southwestern Medical Center; The Hospital for Sick Children; University of Cincinnati College of Medicine; Cincinnati Children’s Hospital Medical Center; Cincinnati Children’s Hospital Medical Center; University of Texas Southwestern Medical Center; University of Texas Southwestern Medical Center; University of Cincinnati College of Medicine; University of Cincinnati College of Medicine; University of Cincinnati College of Medicine

**Keywords:** MMP-7, cholestasis, congenital heart disease, biliary atresia, pulmonary hypertension, biomarker

## Abstract

**Background:**

Matrix metalloproteinase 7 (MMP-7) is a novel biomarker for diagnosis of biliary atresia (BA), the most common cholestatic liver disease in infancy. There is a pressing need to determine the utility of MMP-7 levels in infants with congenital heart disease (CHD) to avoid unnecessary invasive diagnostic procedures in this high-risk population. We investigated the utility of MMP-7 in discriminating BA from non-BA cholestasis in infants with CHD and whether MMP-7 elevation was present in infants requiring treatment for clinically significant PH.

**Methods:**

This is a single center cross sectional study including infants <180 days of age with cholestasis and serum MMP-7 levels collected from 2019–2023. Demographic data and descriptive statistics were summarized with medians with interquartile ranges and frequencies with percentages. Median MMP-7 levels were assessed via Wilcoxon rank-sum test.

**Results:**

A total of 149 patients were included. Patients with CHD had significantly elevated MMP-7 levels relative to the non-CHD cohort (50 vs. 34 ng/mL, p=0.009). Sub-analysis comparing infants with and without PH revealed significantly elevated median MMP-7 levels in those with clinically significant PH (125 vs. 39 ng/mL, p=0.010). CHD patients with PH had greater median MMP-7 compared to CHD patients without PH (154 vs 43 ng/mL, p=0.028).

**Conclusions:**

Serum MMP-7 levels in infants with CHD-C were significantly elevated compared to those with cholestasis alone. MMP-7 may help identify non-BA cholestatic infants who have concurrent clinically significant pulmonary hypertension. Larger, prospective studies are needed to validate this finding and establish CHD-specific MMP-7 cutoffs.

## Introduction

Biliary atresia (BA) is a progressive infantile cholangiopathy with an estimated incidence of 1 in 10,000–15,000 lives births in the United States.[1] Atretic extrahepatic bile ducts in BA lead to progressive biliary cirrhosis and end-state liver disease which, if left untreated, results in death or liver transplantation by 3 years of age.[2] The hepatoportoenterostomy (HPE) surgery is the only treatment for BA, where the atretic bile ducts are removed and a portion of the small intestine is brought up to the porta hepatis to allow for biliary drainage. The key factor in survival for BA patients is undergoing this surgery as early as possible in order to prolong native liver function.[3] However, early diagnosis of BA in the first 45–60 days of life is challenging due to the broad overlap in clinical and laboratory features of BA with other forms of neonatal cholestasis.

Matrix metalloproteinase-7 (MMP-7) is a newly identified biomarker of BA discovered from a large-scale proteomic study conducted by the Childhood Liver Disease Research Network.[4] Subsequent studies in patient cohorts in Asia, Europe, and North America have reproduced the superior discriminatory power of MMP-7 to differentiate BA from other forms of neonatal cholestasis.[5–9] However, these studies were exclusively conducted in term infants without any congenital anomalies, including congenital heart disease, which limits the broader application of MMP-7 screening. Because timing of cardiac repair can affect BA outcomes after HPE, it is critical to distinguish congenital heart disease (CHD) infants who are cholestatic from isolated CHD from those who may also have concurrent BA.[10,11]

Specifically, patients with congenital heart disease also have cholestasis (CHD-C) as a manifestation of RV hypertension, which can be biochemically indistinguishable from BA. BA can also occur in a systemic form (BA-splenic malformation), in conjunction with heterotaxy, congenital heart disease, and polysplenia amongst other midline defects.[12] To date, there are very few published studies describing the association between MMP-7 levels in neonates and infants with cholestasis and CHD, though some studies in older children and adults have looked at the association of MMP levels and pulmonary hypertension.[13]

We sought to investigate the utility of MMP-7 as a screening biomarker for BA in patients with CHD-C. The primary aim of the study was to quantify serum MMP-7 level expression in neonates and infants with concurrent cholestasis and CHD and to compare those levels with patients who solely had cholestasis. This was done to quantify serum MMP-7 expression in infants with biliary atresia and congenital heart disease. The secondary aim of the study was to identify patterns in cardiac defects in CHD cholestatic infants with elevated serum MMP-7 expression.

## Methods

This is a cross-sectional study at a single large quaternary pediatric hospital examining serum MMP-7 levels, clinical characteristics, and outcomes in neonates and infants with and without congenital heart disease and cholestatic jaundice from 2019 – 2022. MMP-7 was used widely at our center after 2019 as a non-invasive biomarker to aid in the diagnosis of BA in cholestatic infants. The retrospective chart review protocol was approved by the Institutional Review Board of Cincinnati Children’s Hospital Medical Center. In this study, patients were identified in the electronic medical record based on pre-defined criteria. Inclusion criteria in the study included neonates (0–30 days old) and infants (1–6 months old) with cholestasis defined as serum direct bilirubin ≥ 1 mg/dL and with or without a diagnosis of congenital heart disease. All included patients had a serum MMP-7 obtained during their hospitalization or outpatient clinic visit. Cholestatic neonates and infants with or without CHD were excluded if they did not have a serum MMP-7 level obtained.

**Figure 1:**
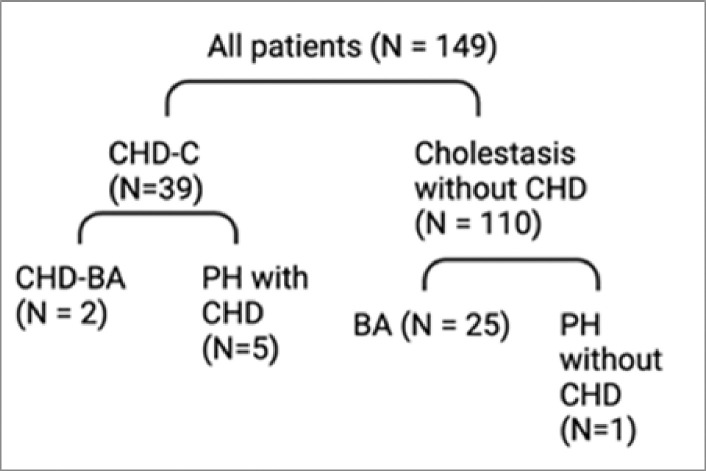
Schematic of patient categories with subgroups.

Laboratory and imaging data collected included: serum transaminases, total and direct bilirubin, gamma glutamyl transferase, INR levels, cholestasis panel genetic testing, total parenteral nutrition administration data, infectious testing, liver biopsy findings, liver ultrasound reports, echocardiography reports, medications, and cardiac catheterization reports where available. Patient characteristics included: age, ethnicity, race, gender, type of cardiac lesion, presence of pulmonary hypertension (PH). Given that not all patients underwent cardiac catheterization, clinically significant PH was defined as requiring the initiation of medical therapy for PH including inhaled nitric oxide, sildenafil, bosentan, and inhaled or intravenous prostacyclin. Patients were grouped into the following categories: cholestasis without CHD with subgroups of BA and neonatal cholestasis (non-BA), and CHD-C with subgroups of pulmonary hypertension (PH) and CHD with biliary atresia (CHD-BA) (see Figure 1).

### Statistical Design

Data were summarized as frequencies (%) for categorical variables and median (interquartile range [IQR]) for continuous variables. Categorical variables were compared using Chi-square or Fishers’ exact tests and continuous variables using Wilcoxon rank-sum test. Comparisons were first made between the neonatal cholestasis and the congenital heart disease groups to assess for differences in demographics, clinical features, and laboratory outcomes. MMP-7 levels were compared between groups including CHD-C vs. cholestasis alone, CHD-BA vs. BA, CHD-C and PH vs. patients without PH, and CHD-C and PH vs. CHD-C alone using Wilcoxon rank sum test. Two-sided p-values <0.05 were considered statistically significant. All statistical analyses were performed using R version 4.3.3 statistical software (R Core Team, 2024), RStudio (RStudio Team, 2024), and the tidyverse package (Wickham, 2017).

## Results

### Patient Characteristics

A total of 149 cholestatic neonates had MMP-7 levels obtained from 2019–2022. 39 patients had CHD-C, and 110 infants had cholestasis (with and without BA). Within the CHD-C group, 2 patients were categorized as CHD-BA. (see Figure 1). 6 patients were in the PH group; 5 had CHD and 1 had PH without structural heart disease.

No significant differences were found in the demographic characteristics between the cholestasis alone and CHD-C groups (Table 1). Serum liver transaminases were not significantly different between groups. Total and direct bilirubin levels were higher in the CHD-C group (Table 2). The CHD-C group had significantly more patients with history of TPN use than the cholestasis alone group (15/38 vs 24/111, p = 0.03); however, the TPN duration did not differ significantly between groups.

### MMP-7 in Cholestasis and Congenital Heart Disease

**Figure 2:**
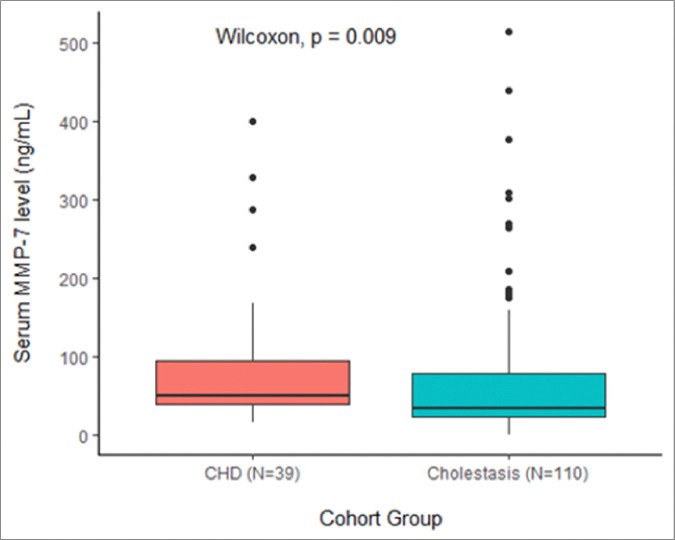
Median MMP-7 levels in patients with CHD-C vs. cholestatic disease alone.

Serum MMP-7 levels were measured in both groups at the time of outpatient hepatology office visit or inpatient hepatology consultation. Median MMP-7 levels were higher in the CHD-C group than the cholestasis alone group [50 ng/mL (IQR 38–95 ng/mL) vs. 34 ng/mL (IQR 23–79 ng/mL), p=0.009] (Figure 2). 27 patients were diagnosed with BA of which two had both CHD and BA (CHD-BA). The CHD-BA patients had an elevated median MMP-7 level of 82 ng/mL – this was above the standard cut-off of for BA of 52.8 ng/mL on the Luminex bead-based assay, but not statistically different from the median MMP-7 level of the BA alone group of 146 ng/mL (IQR 113–210 ng/mL, p=0.09).

### Characterization of Congenital Heart Disease Defects and Elevated Serum MMP-7 Levels

5/38 (13%) CHD-C patients and 1 cholestatic infant without structural heart disease were treated for PH. The five PH patients with structural heart disease included 3 neonates with patent ductus arteriosus, one neonate with double outlet right ventricle and transposition of the great arteries, and one neonate with Ebstein’s anomaly. The neonate without CHD with clinically significant PH had a severe right sided congenital diaphragmatic hernia and omphalocele. All infants were on inhaled nitric oxide and/or sildenafil therapies and diuretics.

**Figure 3:**
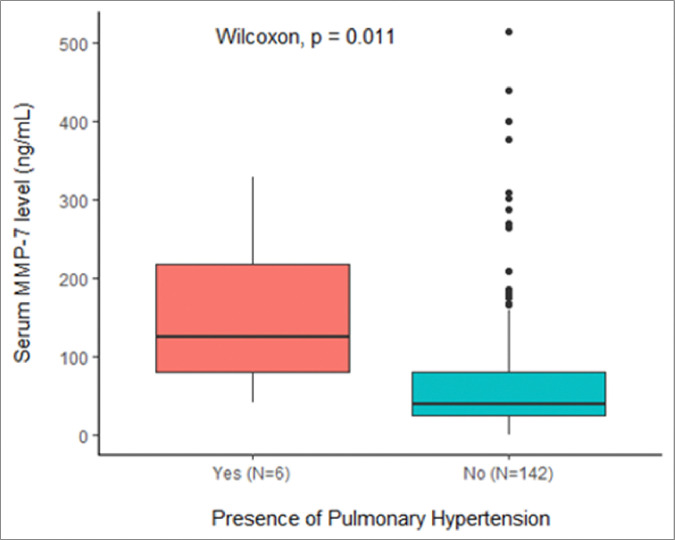
Median MMP-7 levels in patients with PH vs. remaining cohort without PH

**Figure 4:**
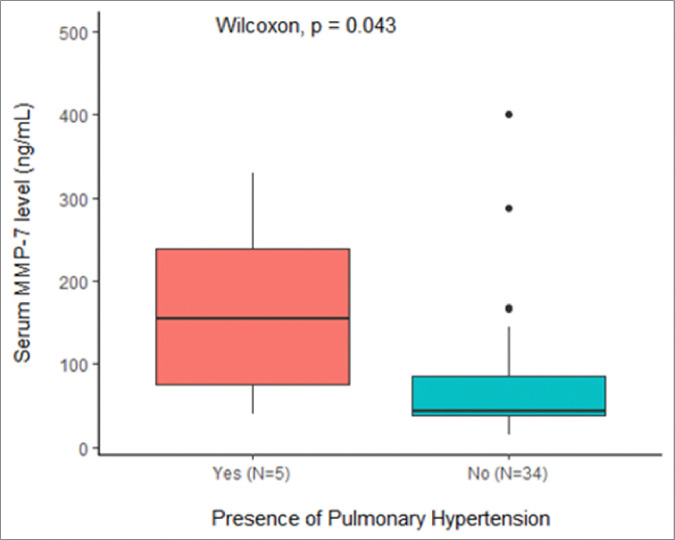
Median MMP-7 levels in patients with CHD-C and PH vs. CHD-C alone.

Median serum MMP-7 levels in these patients were significantly higher than patients without PH (125 ng/mL [IQR 81, 217 ng/mL] vs 39 ng/mL [IQR 25, 77 ng/mL], p=0.01, see Figure 3). Patients with PH had significantly elevated serum MMP-7 levels when compared to the CHD-C only group excluding patients with concurrent BA (154 ng/mL [IQR 76, 238 ng/mL] vs 43 ng/mL [IQR 37–81 ng/mL], p=0.02, see Figure 4).

## Discussion

Our study examined serum MMP-7 expression in infants with co-occurring cholestatic disease and CHD and found significant associations. Patients with CHD-C, particularly those with medically treated PH, had significantly higher MMP-7 levels compared to infants with cholestasis alone. The median MMP-7 level in the CHD-C group was below the established cut-off for BA but was significantly higher than that of the cholestasis alone group. Our data provides valuable insight into the interpretation and potential limitations of MMP-7 in infants with both cholestasis and congenital heart disease. The elevation of MMP-7 in the absence of BA in these groups suggests established cutoff values are not as representative of this population, and current cutoffs should be used cautiously to exclude BA in infants with CHD. As such, further studies are needed to establish MMP-7 utility as a screening biomarker for BA in infants with CHD.

Published studies estimate a prevalence of cholestasis in 18–44% of neonates with CHD-C.[14,15] Concurrent CHD with cholestatic liver disorders, such as Alagille syndrome and choledochal cysts, contributes to a subset of this population.[16–18] Other isolated forms of CHD such as shunt lesions, single ventricular physiology, and valvar stenosis have been associated with cholestasis. CHD combined with cholestasis has additionally been linked with worse cardiac prognosis.[11,14,19,20] It is also purported right-sided cardiac disease (especially pulmonary arterial hypertension) is associated with cholestatic disease, increasing hepatic congestion and affecting hepatic clearance.[21] BA splenic malformation syndrome, a syndromic form of BA, accounts for 10–15% of BA cases, and is associated with a wide spectrum of cyanotic and non-cyanotic CHD types.[22] BA patients with concurrent congenital heart disease are a high risk subgroup of BA, and the timing of cardiac correction prior to hepatoportoenterostomy surgery is critical to improve clinical outcomes.[10]

The biological etiology of MMP-7 may be from both hepatic and extrahepatic sources in this subgroup of patients. Matrix metalloproteinases are involved in tissue repair and extracellular matrix degradation in multiple organs in the human body.[23,24] Specifically, matrix metalloproteinases are also purported to be involved in vascular remodeling and the pathophysiology of pulmonary hypertension development. [25–27] Published studies in adults show that MMP-7 levels are elevated in patients with pulmonary arterial hypertension compared to controls and suggest MMP-7 may play a role in early differentiation of pulmonary artery hypertension from other causes of dyspnea.[28] In older pediatric patients with CHD, serum metalloproteinases have been associated pulmonary arterial stiffness and ventricular dysfunction.[26]

### Study Strengths and Limitations

Strengths of our study include the large cohort of patients with granular biomarker data available to conduct sub-analyses, leading to a better understanding of serum MMP-7 levels in neonates and infants with CHD and the potential association of PH with elevated MMP-7 in infants with CHD. This study is subject to certain limitations. The retrospective single center nature of this study limits its generalizability and should be validated with larger, multicenter cohorts. Additionally, classification of clinically significant pulmonary hypertension in this study was done based on initiation of medical therapy for PH given the very rare number of patients in our study who had a diagnostic cardiac catheterization for PH. Interpretation of the data within the sub-groups must also be cautiously done given there were only two patients with concomitant biliary atresia and congenital heart disease. These limitations notwithstanding, the findings from this study suggest that MMP-7 remains an adequate biomarker for detecting BA in infants with CHD. However, larger, prospective studies are needed to establish CHD-specific MMP-7 cutoffs.

## Conclusions

We demonstrated preliminary evidence that MMP-7 levels indeed can be utilized to discriminate CHD and non-CHD cholestatic disease in neonates. Like prior studies in older children and adults, MMP-7 may also be a promising biomarker of PH in infants and neonates, however, future studies should focus on validation in larger cohorts and on the role of MMP-7 in screening and monitoring of PH in this population.

## Figures and Tables

**Table 1.** Patient demographics of patients with cholestatic disease alone vs. CHD-C.

Characteristic	Neonatal Cholestasis, N = 110^*1*^	Congenital Heart Disease, N = 39^*1*^	P-value^*2*^
Age (mos)	31 (17, 58)	30 (17, 48)	0.834
Sex			0.945
Male	67 (61%)	24 (62%)	
Female	43 (39%)	15 (38%)	
Ethnicity			0.728
Hispanic or Latino	9 (8.5%)	2 (5.1%)	
Non-Hispanic or Latino	97 (92%)	37 (95%)	
Race			0.381
White	70 (67%)	21 (54%)	
Black	25 (24%)	13 (33%)	
Asian	3 (2.9%)	1 (2.6%)	
Other/mixed	6 (5.8%)	4 (10%)	

**Table 2.** Clinical and laboratory data of patients with cholestatic disease alone vs. CHD-C.

Characteristic	Neonatal Cholestasis, N = 110^*1*^	Congenital Heart Disease, N = 39^*1*^	p-value^*2*^
ALT (U/L)	59 (28, 120)	71 (37, 175)	0.561
AST (U/L)	91 (44, 193)	114 (73, 240)	0.222
Total bilirubin (mg/dL)	7.1 (3.9, 10.8)	9.7 (6.2, 13.0)	0.034
Direct bilirubin (mg/dL)	2.7 (1.2, 5.1)	5.8 (3.1, 8.1)	<0.001
GGT (U/L)	172 (86, 409)	111 (56, 339)	0.124
INR	1.08 (0.98, 1.20)	1.14 (1.06, 1.28)	0.051
TPN use (%)	24 (22%)	15(38%)	0.045
TPN duration (days)	17 (7, 27)	19 (12, 31)	0.449
Prematurity	29 (27%)	17 (44%)	0.053
